# IMU Auto-Calibration Based on Quaternion Kalman Filter to Identify Movements of Dairy Cows

**DOI:** 10.3390/s24061849

**Published:** 2024-03-13

**Authors:** Carlos Muñoz-Poblete, Cristian González-Aguirre, Robert H. Bishop, David Cancino-Baier

**Affiliations:** 1 Electrical Engineering Department, Universidad de La Frontera, Temuco 4811230, Chile; 2 Magister en Ciencias de la Ingeniería, Universidad de La Frontera, Temuco 4811230, Chile; 3 Electrical Engineering, University of South Florida, 4202 E. Fowler Ave., Tampa, FL 33620, USA; 4 Facultad de Ciencias Agropecuarias y Medioambiente, Universidad de La Frontera, Temuco 4811230, Chile

**Keywords:** self-calibration, Kalman filter, cow attitude estimation, IMU-based collar

## Abstract

This work is focused on developing a self-calibration algorithm for an orientation estimation of cattle movements based on a quaternion Kalman filter. The accelerometer signals in the earth’s frame provide more information to confirm that the cow is performing a jump to mount another cow. To obtain the measurements in the earth’s frame, we propose a self-calibration method based on a strapdown inertial navigation system (SINS), which does not require intervention by the user once deployed in the field. The self-calibration algorithm uses a quaternion-based Kalman filter to predict the angular orientation with bias correction, and update it based on the measurements of accelerometers and magnetometers. The paper also depicts an alternate update to adjust the inclination using only the accelerometer measurements. We conducted experiments to compare the accuracy of the orientation estimation when the body moves similarly to cow mount movements. The comparison is between the proposed self-calibration algorithm with the IvenSense *MPU9250* and Bosch *BNO055* and the quaternion attitude estimation provided in the *BNO055*. The auto-calibrating algorithm presents a mean error of 0.149 rads with a mean consumption of 308.5 mW, and the Bosch algorithm shows an average error of 0.139 rads with a mean consumption of 307.5 mW. When we executed this algorithm in an *MPU9250*, the average error was 0.077 rads, and the mean consumption was 277.7 mW.

## 1. Introduction

Timely and accurate estrus detection is a long-standing problem affecting dairy producers [1]. The non-detection, or discrimination, of events of interest that allow the detection of estrus, results in an increase in the interpartum period, which translates into losses in milk production and in the efficiency of reproductive management; that is, the loss of money for the production system [2].

According to Göncü and Koluman [3], errors in estrus detection can cost between USD 2 and 6 per extra day in the interpartum period, and the loss of one estrous cycle (21 days) could cost between USD 42 and 126 per cow. The efficiency in milk production is related to the liters produced daily by a cow and the number of days that it is lactating. This number of days increases as the period between delivery and the following service “open days” decreases, which conventionally is around 60 days [4]; therefore, an increase in this period decreases the annual milk production. To maintain the open days period within the 60-day range, cows must become pregnant as soon as possible, and in that sense, a return to a normal estrous cycle and early estrus detection is essential. According to Mičiaková et al. [5] and Brehme et al. [6], estrus could last between 7.5 and 9.5 h, varying among different breeds, 10/12 h after the ovulation begins. Thus, detecting estrus is essential as it is the only signal farmers have to know when ovulation occurs, and when it is necessary to inseminate or allow mating.

In recent years, the aging of dairy workers and the lack of skilled labor in rural sectors, as explained by Hirata et al. [7], has led to a decrease in the efficiency of visual detection of the heat of around 55% to 70% in just 20 years, forcing the production system to seek more efficient detection methods. Another critical point considered by Hirata et al. [7] is that estrus expresses in approximately the same proportion during the day and night, the latter period being even more complex to perform a visual detection. Today, the most commonly used methods for this purpose, from the most practical to the most precise, such as the use of patches accompanied by visual inspection (which requires time and adequate experience), vasectomized bulls, andronized cows, and clinical techniques, such as ultrasonography, progesterone detection in milk, and observation of vaginal secretions [5].

Automatic techniques that significantly reduce the personnel needed for this labor have been introduced recently. These techniques use pedometers, accelerometers, mount detectors, and ruminal monitors [5] as detection mechanisms, all associated with the animal’s behavior and activity. Recently, they have been complemented with the use of real-time video surveillance [7].

Activity identification is the most used mechanism to detect cows in heat automatically; however, there is no uniform way to measure activity using accelerometer-based collars. For example, Løvendah and Chagunda [8] used electronic activity tags fitted on neckbands (Alpro, version 6.60; DeLaval, 2007). An activity count represents the average activity level within the selected interval, which can be set from 2 s to 15 min. Mayo et al. [9] performed an analysis of different commercial devices that measure the activity in steps, neck movement, high activity of head movement, or a proprietary motion index (that refers to an activity index developed by the vendor), which increased on the day of estrus from 69% to 170% when compared to the baseline before estrus. Although automatic detection methods based on activity are an excellent alternative to visual inspection systems due to the lower labor requirement, their success rates do not exceed 70% [5].

Pfeiffer et al. [10] compared the evaluation of different devices, showing that some of them reach up to a 80% success rate only when they are complemented with other measurements. The results obtained by Nelson et al. [11] with Hereford cows showed that detection with mechanisms based on activity measurement reached 68%, while those based on visual inspection reached 89%. Visual inspection is based on a scale established by Van Eerdenburg et al. [1], which assigns scores to behaviors detected by visual observations. For example, he assigned 100 points when a cow allows itself to be mounted by another cow, and for the cow’s activity status, he only assigned 5 points. In this case, the activity status is defined as the percentage of time the cow is walking or jogging. One way to identify when a cow allows itself to be mounted is by using marker patches on the animal’s tail-head. This is confirmed by Hill et al. [12], who showed with an accuracy of more than 90% that the patches are effective in identifying the most suitable time to carry out artificial insemination to improve the fertility rate. It should be noted that this procedure requires personnel’s assistance to verify that the patches are not stained and to apply them again if needed. There are also pressure-sensitive adhesive devices, such as FlashMate [13], which identifies cows in heat when the contacts’ frequency exceeds a threshold within a variable time window in the previous hours. This device does not transmit data wirelessly, but produces a blinking visual signal when activated [14,15]. On the other hand, there are patches with pressure detectors and electronic transmitters, presenting an effectiveness of 56–94% [5].

The automatic identification of movements (like mounts) is difficult with data acquired from a collar located around the neck of the cow because when the cow jumps to mount, the head is oriented in the direction the cow intends to move. The accelerometer signals provided in the three axes are taken in the frame of a cow body, so it is difficult to discriminate which acceleration offers more information to confirm that the cow is performing a jump to mount another cow.

There is evidence in the published literature, in which movement classification is performed using raw data from the IMU for calibration. For example, the article [16] focuses on the study of sheep for the purpose of classifying their movements. These movements, subject to measurement, are influenced by the orientation that the IMU adopts as the movement is carried out. Therefore, the movement pattern which is being attempted to classify could experience significant variations depending on the positioning of the sheep’s neck. Another problem with using raw data is that the IMUs provide this information with different scaling, so if you change from an IMU manufactured by one supplier to one manufactured by a different supplier, the calibration model changes because the parameters obtained from the classifier depend on the type of data used for training. This is relevant since product changes due to the technological obsolescence of the IMUs can lead to the need to collect mount signals for training again, which is a long and complex process [17]. However, Liu et al. [18] proposes a processing stage for the measured signals that consist of obtaining modules from the raw data, followed by a classification system based on neural networks. The author’s methodological approach to measured data deviates from the identification of more complex movements as it loses orientation information. Despite this, it manages to obtain good results in measuring activity indices. Andriamandroso et al. [19] goes one step further and proposes a pre-processing stage based on the normalization of measurement signals. The authors used a smartphone as a capture device in the collar of the cow, so they had already calibrated data and orientation provided by the device. They used the collected data only for analytical purposes and as support for the classification of movements, but not to make orientation updates.

The alternative is to use the normalized accelerations taken by the collar but expressed in the earth’s frame of reference. In this sense, when the cow attempts to mount, the acceleration in the *z* axis is easily distinguishable from the other accelerations, in particular, when the cow falls down to a standing position. To obtain the accelerations in the world frame of reference, the accelerations acquired in the body frame must be rotated using the orientation of the collar expressed in the world frame of reference. Something similar happens when trying to identify walking from accelerations measured in the body’s frame. When the cow walks, there are accelerations in the forward and backward sense in the plane of the earth. In this case, the module of the acceleration in the (x,y) plane, in the world’s frame, is more helpful in discriminating the walking movements.

As the IMU is strapped to the cow’s collar, to obtain the attitude in the world’s frame, we need to solve the problem known as strapdown inertial navigation systems (SINS), which is a method to determinate the position, speed, and orientation of moving objects without external information [20,21]; Chang and Li [22] showed that skew errors are better handled using quaternions to design attitude algorithms. Zhong et al. [23] proved that the quaternion-based nonlinear models contain high-order error items, which allow the accurate description of the nonlinear characteristics of the SINS navigation system and the variation of attitude errors. The use of Kalman filters is also reported in biological characterization, Zhu [24] employed extended Kalman filters for the real-time soft tissue characterization of robotic-assisted minimally invasive surgery, achieving precise haptic control of robotic surgical tasks and providing realistic force feedback to the operator. In the case of application to biological systems, Zhang et al. [25] presented a standing calibration method of the MEMS gyro bias for autonomous pedestrian SINS when the human body sways slightly. The methods do not use external measurements for calibration, and they show in a turntable that the errors of the auto-calibrating method were accurate.

Usually, the attitude estimation is only available in some middle-cost IMUs, such as Bosch *BNO055*, but less expensive IMUs do not provide reliable methods to obtain the attitude. The challenge addressed in this article is to obtain an accurate measurement of the attitude of the cow´s IoT collar using low-cost IMU and using as low a computational load as possible in order to drain the minimum amount of energy from the onboard batteries.

## 2. Initial Calibration Models

Although the IMUs are meant to provide acceleration, gyroscope, and magnetometer data, they are very different and have unique properties due to the fabrication procedure used by the vendor, furthermore, the Bosch *BNO055* and the InvenSense *MPU9250* provides the raw data in different formats. It is advisable to use an initial calibration model to compare the measurements. A simple method based on collecting measurements of the Earth’s magnetic field and the field of gravity accelerations, according to [26] is used in this work.

### 2.1. Initial Accelerometer Calibration Model

Consider the model,

(1) $$a_{c}\left(i\right)=K_{a}S_{a}\left(a_{m}\left(i\right)+b_{a}+n_{a}\right),\;i=1,\cdots ,n$$

where $a_{c}$ and $a_{m}$ are the calibrated acceleration and the measured acceleration, respectively, and the parameters of the calibration matrices $K_{a}$ (scaling matrix), $S_{a}$ (Non-orthogonal matrix), and $b_{a}$ (bias vector) are given by the following:

(2) $$K_{a}=\left[\begin{array}{ccc} k_{x,a} & 0 & 0\\ 0 & k_{y,a} & 0\\ 0 & 0 & k_{z,a} \end{array}\right]\;S_{a}=\left[\begin{array}{ccc} 1 & \gamma_{xz,a} & \gamma_{xy,a}\\ \gamma_{yz,a} & 1 & \gamma_{yx,a}\\ \gamma_{zy,a} & \gamma_{zx,a} & 1 \end{array}\right]\;b_{a}=\left[\begin{array}{c} b_{x,a}\\ b_{y,a}\\ b_{z,a} \end{array}\right]$$

and $n_{a}$ is modeled as a white noise with zero mean and variance given by the following:

(3) $$\begin{array}{c} Var\left(n_{a}\right)=\left[\begin{array}{c} \sigma_{x,a}^{2}\\ \sigma_{y,a}^{2}\\ \sigma_{z,a}^{2} \end{array}\right] \end{array}$$

When the body containing the accelerometer is steady, the magnitude of the measured acceleration must be 1*g* (*g* = 9.8 m/s), and to correct the scaling, the non-orthogonality, and the bias in (2), it is advisable to take *n* measurements of $a_{m}\left(i\right)$ with the body containing the accelerometer aimed at different orientations. This involves finding the values of the parameter vector $\theta_{a}=[k_{x,a},\;k_{y,a},\;k_{z,a},\;\gamma_{xz,a},\;\gamma_{xy,a},\;\gamma_{yz,a},\;\gamma_{yx,a},\;\gamma_{zy,a}$, $\gamma_{zx,a},\;b_{x,a},\;b_{y,a},\;b_{z,a}]$, which minimize the error between a function dependent on the calibration parameters of the measurement model. This accelerometer model is calibrated in the laboratory using the cost function (4), according to the calibration procedure depicted in Bonnet et al. [27].

(4) $$\begin{array}{c} \theta_{a}=arg\min_{\theta_{a}}\left\{J_{a}=\sum_{i=1}^{n}e_{a}\left(i\right)\right\} \end{array}$$

with the error $e_{a}\left(i\right)$ given by the following:

(5) $$\begin{array}{c} e_{a}\left(i\right)=\sum_{i=1}^{n}{\left(\left|\right|a_{c}\left(i\right)\left|\right|-1\right)}^{2},\;i=1,\cdots ,n \end{array}$$

### 2.2. Initial Magnetometer Calibration Model

Similarly to the accelerometer, the model for the magnetometer measurement is given by Equation (6),

(6) $$B_{c}\left(i\right)=K_{b}S_{b}\left(B_{m}\left(i\right)+b_{b}+n_{b}\right),\;i=1,\cdots ,n$$

where $B_{c}$ and $B_{m}$ are the calibrated magnetic field and the measured magnetic field, respectively, and the calibration matrices $K_{b}$ (scaling matrix), $S_{b}$ (non-orthogonal matrix), and $b_{b}$ (bias vector), these matrices include the interference given by hard iron, which is modeled in the biases in the calibration model, while the interference of soft iron is modeled through the scaling factors [28] and are given by the following:

(7) $$K_{b}=\left[\begin{array}{ccc} k_{x,b} & 0 & 0\\ 0 & k_{y,b} & 0\\ 0 & 0 & k_{z,b} \end{array}\right]\;S_{b}=\left[\begin{array}{ccc} 1 & \gamma_{xz,b} & \gamma_{xy,b}\\ \gamma_{yz,b} & 1 & \gamma_{yx,b}\\ \gamma_{zy,b} & \gamma_{zx,b} & 1 \end{array}\right]\;b_{b}=\left[\begin{array}{c} b_{x,b}\\ b_{y,b}\\ b_{z,b} \end{array}\right]$$

and $n_{b}$ is modeled as a white noise with zero mean and variance given by the following:

(8) $$\begin{array}{c} Var\left(n_{b}\right)=\left[\begin{array}{c} \sigma_{x_{b}}^{2}\\ \sigma_{y_{b}}^{2}\\ \sigma_{z_{b}}^{2} \end{array}\right] \end{array}$$

The calibration methodology for the magnetometer is similar to that of the accelerometer, and, in this case, the measurements are normalized in order to find the magnetic North, so, the magnitude of the magnetic field must be one. The measurements are taken in various orientations to estimate parameters such as scaling, non-orthogonality, and bias. The objective is to minimize the disparity between actual magnetic field measurements (9) and the expected values based on the following parameters: $\theta_{b}=[k_{x,b},\;k_{y,b},\;k_{z,b},\;\gamma_{xz,b},\;\gamma_{xy,b},\;\gamma_{yz,b},\;\gamma_{yx,b}$, $\gamma_{zy,b},\;\gamma_{zx,b},\;b_{x,b},\;b_{y,b},\;b_{z,b}]$.

(9) $$\begin{array}{c} e_{b}\left(i\right)=\sum_{i=1}^{n}{\left(\left|\right|B_{c}\left(i\right)\left|\right|-1\right)}^{2},\;i=1,\cdots ,n \end{array}$$

This process is conducted in a laboratory environment and utilizes a cost function, similar to the accelerometer calibration, given by the following:

(10) $$\begin{array}{c} \theta_{b}=arg\min_{\theta_{b}}\left\{J_{b}=\sum_{i=1}^{n}e_{b}\left(i\right)\right\} \end{array}$$

### 2.3. Magnetic Tilt Correction

The existence of a magnetic field component perpendicular to the earth’s surface generates problems when attempting to estimate the orientation, since the basic principles on which the self-calibration algorithm is based, we first use the magnetic field vector perpendicular to the gravity vector as a reference. To solve this problem, we calculate and separate the horizontal component of the magnetic field at the earth’s surface. The method of projection of the magnetic field vector onto the gravity acceleration vector is given by the magnetometer.

(11) $$proy\left({\vec{B}}_{c,\vec{a}}\right)=\frac{{\vec{B}}_{c}\cdot {\vec{a}}_{c}}{\left|\right|{\vec{a}}_{c}\left|\right|}{\vec{a}}_{c}$$

Equation (11) will be used to estimate the perpendicular component and it will be subtracted from the calibrated measurement given by the following:

(12) $${\vec{B}}_{c}^{*}={\vec{B}}_{c}-proy\left({\vec{B}}_{c,\vec{a}}\right)$$

In this way, it is possible to eliminate the magnetic field component perpendicular to the earth’s surface using the readings of the magnetometer and accelerometer and, thus, we are only left with the horizontal component, which will be used as a reference to perform the IMU self-calibration. However, when eliminating a component of the magnetic field vector, it is necessary to normalize the new vector obtained as follows:

(13) $$\vec{B}=\frac{{\vec{B}}_{c}^{*}}{\left|\right|{\vec{B}}_{c}^{*}\left|\right|}$$

which will be aligned to the tangent plane to the earth’s surface, and will be perpendicular to the gravity vector.

### 2.4. Gyroscope Scaling

To keep the procedure as simple as possible, we scale the gyroscope to convert the raw values into rad/sec units using the factors informed by the vendors in the IMUs *BNO055* [29] and *MPU9250* [30] datasheets. The procedure assumes that the most significant error of the gyroscope is in the bias parameters, and the KF can correct this error.

## 3. Attitude Auto-Calibration

It is possible to obtain accurate measurements of the attitude of the cow´s collar in the world frame using a simplified SINS approach, considering that most of the time, the cows perform slow movements, like grazing, ruminating, or resting. The first step is to obtain the orientation of the cow’s collar, i.e., body frame, and make a prediction of the attitude and correct the measurements of the accelerometer and magnetometer in the world frame, and compare it with the expected ones if the cow is not moving. A practical method to obtain the orientation of the body frame is to integrate the angular velocity measured by the gyroscope. However, this mechanism must deal with a time-varying bias inherent to the measurement process of the IMU. One approach is to use a Kalman filter, an algorithm based on a system’s state variables model. Friedland [31] pioneered the application of the Kalman filter utilizing quaternions, a development that has since evolved into a widely adopted technique for strapdown inertial navigation systems (SINS) [22,32,33]. In the present study, we employ the Kalman filter approach for model prediction and update, addressing two distinct scenarios for the update. The first involves updating the model using acceleration and magnetometer measurements, while the second focuses solely on updating with accelerometer data.

### 3.1. Model Prediction

The system model starts from the equation.

(14) $$\dot{\bar{q}}=\frac{1}{2}\left[\bar{q}⊗{\bar{w}}^{b}\right]=\frac{1}{2}\left[{\bar{w}}^{b}⊗\bar{q}\right]$$

where $\bar{q}$ is the attitude quaternion, composed of a scalar term, $q_{0}$ and three imaginary terms, $q_{1},q_{2}$, and $q_{3}$, and ${\bar{w}}^{b}$ is the quaternion for the angular velocity in the body reference as follows:

(15) $$\bar{q}=\left[\begin{array}{c} q_{0}\\ q_{1}\\ q_{2}\\ q_{3} \end{array}\right],\;{\bar{w}}^{b}={\left[\begin{array}{c} 0\\ w \end{array}\right]}^{b}$$

The angular velocity is modeled by the quaternion, ${\bar{w}}^{b}$, given by the following:

(16) $$\bar{w}=\bar{u}+\bar{b}$$

where $\bar{u}$ is the gyroscope measurement quaternion (17) and $\bar{b}$ (18), is a quaternion whose components are the bias of the angular velocity measurement, as shown in (16). This bias $\bar{b}$ is the variable to be estimated in real-time.

(17) $$\bar{u}={\left[\begin{array}{c} 0\\ u \end{array}\right]}^{b},\;u={\left[u_{x},u_{y},u_{z}\right]}^{T}$$

(18) $$\bar{b}={\left[\begin{array}{c} 0\\ b \end{array}\right]}^{b},\;b={\left[b_{x},b_{y},b_{z}\right]}^{T}$$

From (14)–(16), the derivative of a quaternion is as follows:

(19) $$\dot{\bar{q}}=\frac{1}{2}\left[\left(\bar{u}+\bar{b}\right)⊗\bar{q}\right]=\frac{1}{2}\left[\bar{u}⊗\bar{q}\right]+\frac{1}{2}\left[\bar{b}⊗\bar{q}\right]$$

Considering the following approximation:

(20) $$\dot{\bar{q}}=\frac{{\bar{q}}_{k}-{\bar{q}}_{k-1}}{T}$$

where *T* represents the sampling time, then, from (19) and (20); thus, the following:

(21) $${\bar{q}}_{k}={\bar{q}}_{k-1}+\frac{T}{2}\left[{\bar{u}}_{k-1}⊗{\bar{q}}_{k-1}\right]+\frac{T}{2}\left[{\bar{b}}_{k-1}⊗{\bar{q}}_{k-1}\right]$$

where we assume that the bias $\bar{b}$ is constant between $T_{k-1}$ and $T_{k}$ as follows:

(22) $$b_{k}=b_{k-1}$$

Following the work of Li [34], we define the state vector $x_{k}$ as follows:

(23) $$x_{k}={\left[\begin{array}{c} \bar{q}\\ b \end{array}\right]}_{k}={\left[\begin{array}{ccccccc} q_{0} & q_{1} & q_{2} & q_{3} & b_{x} & b_{y} & b_{z} \end{array}\right]}_{k}^{T}$$

This model allows you to correct the angles provided by the angular velocity model, auto-calibrating the parameter bias to compensate for the gyroscope drifts in real-time. In this case, the calibration is only made with the data measured by the IMU. This process is an auto-calibration because the SINS algorithm adjusts the gyroscope bias in real-time without external measurements. The auto-calibration is relevant because once the collars are deployed with the IMU device strapped in the cow collar, we need to keep updating the correction of the gyro bias while the cows are in the grassland. We use the Quanser platform only to verify the goodness of this correction and the energy consumption.

Then, Equations (21) and (22) can be expressed as follows:

(24) $${\hat{x}}_{k}^{-}=A_{k-1}{\hat{x}}_{k-1}+B_{k-1}u_{k-1}$$

where $u_{k-1}$ is the gyroscope measurement at time k−1; thus, the following:

(25) $$\begin{array}{cc} S_{k}={\left[\begin{array}{ccc} -q_{1} & -q_{2} & -q_{3}\\ q_{0} & -q_{3} & q_{2}\\ q_{3} & q_{0} & -q_{1}\\ -q_{2} & q_{1} & q_{0} \end{array}\right]}_{k}\; & \begin{array}{ccc} \; & A_{k}= & {\left[\begin{array}{cc} I_{4\times 4} & -\frac{T}{2}S\\ 0_{3\times 4} & I_{3\times 3} \end{array}\right]}_{k}\\ \; & B_{k}= & {\left[\begin{array}{c} \frac{T}{2}S\\ 0_{3\times 3} \end{array}\right]}_{k} \end{array} \end{array}$$

This is also known as a priori estimate, ${\hat{x}}_{k}^{-}$. Note that in (24), the matrices $A_{k}$ and $B_{k}$ are evaluated at each time *k* using the estimated attitude $q_{k}$. Considering a sample time in the order of 10 times per second, the variation of the attitude $q_{k}$ due to the cow’s slow movements is negligible, so we assumed that these matrices are almost constant between two consecutive time samples, and we opted to keep the Kalman filter structure instead of an extended Kalman filter.

### 3.2. Measurement Update with Accelerometer and Magnetometer

To obtain the a posteriori state, ${\hat{x}}_{k}$, the Kalman filter [35] uses a correction by comparing the measured output $y_{k}^{b}$ with an inferred output ${\hat{y}}_{k}^{b}$ based on the a priori state ${\hat{x}}_{k}^{-}$, given by the following:

(26) $${\hat{x}}_{k}={\hat{x}}_{k}^{-}+K_{k}\left(y_{k}^{b}-{\hat{y}}_{k}^{b}\right)$$

In this case, the selected outputs to be compared are the magnetometers and accelerometers measured and inferred in the frame of reference located in the sensing device, where also the body reference is as follows [34]:

(27) $$y_{k}^{b}={\left[\begin{array}{c} a^{b}\\ m^{b} \end{array}\right]}_{k}\;\mathrm{and}\;{\hat{y}}_{k}^{b}={\left[\begin{array}{c} {\hat{a}}^{b}\\ {\hat{m}}^{b} \end{array}\right]}_{k}$$

In this case, we assume the magnetic field vector with coordinates in the reference frame of the world is ${\hat{m}}_{k}^{w}=\left[0,1,0\right]$, assuming the cows show scarce movements. The acceleration magnitude shown in these movements is usually lower than the gravity acceleration; therefore, we will assume these cow accelerations are negligible with respect to the gravity acceleration, so we will compare the acceleration measured in the IMU only with the dominant acceleration (gravity) given in the world reference, ${\hat{a}}_{k}^{w}=\left[0,0,1\right]$.

To compare these outputs in the body frame of reference, we use the matrix rotation, $R_{w}^{b}$, for the quaternion $\bar{q}$. In this matrix, the superscript *b* symbolizes the body’s frame of reference and the subscript *w* symbolizes the world’s frame of reference, hence the following:

(28) $$R_{w}^{b}=\left[\begin{array}{ccc} q_{0}^{2}+q_{1}^{2}-q_{2}^{2}-q_{3}^{2} & 2(q_{1}q_{2}+q_{0}q_{3}) & 2(q_{1}q_{3}-q_{0}q_{2})\\ 2(q_{1}q_{2}-q_{0}q_{3}) & q_{0}^{2}-q_{1}^{2}+q_{2}^{2}-q_{3}^{2} & 2(q_{2}q_{3}+q_{0}q_{1})\\ 2(q_{1}q_{3}+q_{0}q_{2}) & 2(q_{2}q_{3}-q_{0}q_{1}) & q_{0}^{2}-q_{1}^{2}-q_{2}^{2}+q_{3}^{2} \end{array}\right]$$

This matrix $R_{w}^{b}$ allows us to express the gravity acceleration given in the world reference, $a^{w}=\left[0,0,1\right]$, as follows:

(29) $${\hat{a}}_{k}^{b}=R_{w}^{b}{\hat{a}}_{k}^{w}=R_{w}^{b}{\left[\begin{array}{c} 0\\ 0\\ 1 \end{array}\right]}^{w}=-\left[\begin{array}{c} 2(q_{1}q_{3}-q_{0}q_{2})\\ 2(q_{2}q_{3}+q_{0}q_{1})\\ q_{0}^{2}-q_{1}^{2}-q_{2}^{2}+q_{3}^{2} \end{array}\right]=C_{k}^{a}{\bar{q}}_{k}$$

To express the gravity acceleration from the world frame in the body frame, multiplying the rotation matrix by the attitude quaternion is required, resulting in a non-linear equation; therefore, it is necessary to define a matrix $C_{a}$ expressed in terms of the quaternion $\bar{q}$, as follows:

(30) $$\begin{array}{c} C_{k}^{a}=-{\left[\begin{array}{cccc} -q_{2} & q_{3} & -q_{0} & q_{1}\\ q_{1} & q_{0} & q_{3} & q_{2}\\ q_{0} & -q_{1} & -q_{2} & q_{3} \end{array}\right]}_{k} \end{array}$$

For the magnetometer, we have the rotation matrix based on quaternions, as stated in Equation (28), and already assigned the magnetic field vector with coordinates in the world reference frame, $m_{k}^{w}=\left[0,1,0\right]$, as follows:

(31) $${\hat{m}}_{k}^{b}=R_{w}^{b}{\left[\begin{array}{c} 0\\ 1\\ 0 \end{array}\right]}^{w}={\left[\begin{array}{c} 2(q_{1}q_{2}+q_{0}q_{3})\\ q_{0}^{2}-q_{1}^{2}+q_{2}^{2}-q_{3}^{2}\\ 2(q_{2}q_{3}-q_{0}q_{1}) \end{array}\right]}_{k}=C_{k}^{m}{\bar{q}}_{k}$$

As with the estimation of the acceleration, Equation (31) is nonlinear; therefore, it is necessary to factor by a matrix $C_{m}$ based on the quaternion $\bar{q}$ to compare it with the measured acceleration in the body frame. To fulfill (31), $C_{m}$ is represented as follows:

(32) $$\begin{array}{c} C_{k}^{m}={\left[\begin{array}{cccc} q_{3} & q_{2} & q_{1} & q_{0}\\ q_{0} & -q_{1} & q_{2} & -q_{3}\\ -q_{1} & -q_{0} & q_{3} & q_{2} \end{array}\right]}_{k} \end{array}$$

Finally, using $C_{a}$ and $C_{m}$, the inferred output ${\hat{y}}_{k}^{b}$ is as follows (33):

(33) $${\hat{y}}_{k}^{b}={\left[\begin{array}{c} {\hat{a}}^{b}\\ {\hat{m}}^{b} \end{array}\right]}_{k}=\left[\begin{array}{cc} C_{k}^{a} & 0_{3x3}\\ C_{k}^{m} & 0_{3x3} \end{array}\right]{\left[\begin{array}{c} \bar{q}\\ b \end{array}\right]}_{k}=C_{k}{\hat{x}}_{k}^{-}$$

### 3.3. Measurement Update Only with Acceleration

In our application, we do not require precision in the orientation of the cow, with respect to the north or the east, but we require precision with respect to the inclination of the device because if the cow’s head is aimed to the ground or up to another cow, the acceleration measured in the body reference will be difficult to interpret as a cow’s walking movement or a cow’s mount movement. The correct tilt angle allows a ground-based representation of the accelerations to analyze the cow’s movements. This auto-calibration shows the same benefits as the full attitude calibration to identify a cow’s movements, but the energy requirements are lower. It spares the use of the magnetometer and requires fewer computations.

The inclination auto-calibration is simpler because it only uses the measurements of acceleration and gyroscopes, dismissing the use of the magnetometer. This is relevant because the magnetometer power consumption is the most consuming measurement in the IMU [30]. In this case, the outputs to be compared are the accelerometers measured and inferred in the frame of reference located (34) in the sensing device, also named body reference as follows:

(34) $$y_{k}^{b}={\left[\begin{array}{c} a^{b} \end{array}\right]}_{k}\;\mathrm{and}\;{\hat{y}}_{k}^{b}={\left[\begin{array}{c} {\hat{a}}^{b} \end{array}\right]}_{k}$$

where the inferred output ${\hat{y}}_{k}^{b}$ is (35).

(35) $${\hat{y}}_{k}^{b}={\left[\begin{array}{c} {\hat{a}}^{b} \end{array}\right]}_{k}=\left[\begin{array}{cc} C_{k}^{a} & 0_{3x3} \end{array}\right]{\left[\begin{array}{c} \bar{q}\\ b \end{array}\right]}_{k}=C_{k}{\hat{x}}_{k}^{-}$$

### 3.4. Status Update

To solve Equation (26), we need Equation (36), to update the a priori covariance, to update the Kalman gain, $K_{k}$, and to update the a posteriori covariance matrix, $P_{k}$, via [35],

(36) $$\begin{array}{c} {\hat{P}}_{k}^{-}=A_{k}P_{k-1}A_{k}^{T}+Q_{n} \end{array}$$

(37) $$\begin{array}{c} K_{k}=P_{k}^{-}C_{k}^{T}{\left[C_{k}P_{k}^{-}C_{k}^{T}+R_{n}\right]}^{-1} \end{array}$$

(38) $$\begin{array}{c} P_{k}=\left(I-K_{k}C_{k}\right)P_{k}^{-} \end{array}$$

## 4. Experimental Setup

The IoT collar comprises an IMU and a Heltec ESP32 microcontroller with LoRaWAN. The IMUs used are the Bosch *BNO055* and the InvenSense *MPU9250*, both with three-axis accelerometers, magnetometers, and gyroscopes. *BNO055* is more expensive and came with a quaternion estimate of the attitude provided in the firmware. We will execute the developed auto-calibration algorithms in real-time and compare them in terms of attitude estimation and power consumption. An experimental board of the IoT collar was constructed for this comparison. The board integrates a Heltec wireless stick module as a main processor and two IMUs, an InvenSense *MPU9250*, and a Bosch *BNO055* connected through $I^{2}C$ bus. A clock configuration for the $I^{2}C$ protocol was implemented with an operating frequency set to 100 kHz in the Arduino IDE environment. The sensors have been configured to operate within the same operational range, ensuring that they sample synchronously at a rate of 10 Hz. To accurately track the cow’s movements, the *BNO055* and *MPU9250* sensors were meticulously configured. The gyroscope measurement range for both sensors was set to +/− 2000 DPS, while the accelerometer range was set to +/− 2G. Specifically, for the *BNO055*, it was operated using the “fusion mode/NDOF” mode to estimate the quaternion attitude. This algorithm is provided “as is” within the *BNO055*, and it does not require additional setup.

### 4.1. Initial Calibration Procedure

The initial calibration does not require reference measurements for the initial calibration procedure (see Section 1). For this procedure, the experimental board must be kept resting in a set of fixed directions spaced in 3D. With the tests, we found that 30 regularly spaced measurements provided enough information to proceed with the calibration. According to our experiments, the contribution to the goodness of the fit is marginal when the number of evenly spaced positions in 3D space increases over 30. To ensure the device was still in each new position, we checked the stationary position of the gyroscope measurements. When the gyroscope module reaches a steady state for each of these positions, we acquire the complete set of measurements (accelerometers and the magnetometer in 3D). With the data collected, we will apply the initial calibration depicted in Section 2.1 to calibrate the accelerometer model (1) and the magnetometer model (6).

### 4.2. Testing Procedure

The Kalman filter algorithms shown in Section 2 do not require external measurements to calibrate the parameters of the model, as they use the magnetic and gravity fields to make the corrections. The Kalman filter updates in real-time, not only to the initial conditions of the angles, but also to the bias parameters of the model (19), correcting the drift of the gyroscope. Prior to putting the collars on the cows, we will use an experimental setup consisting of a Quanser platform to emulate movements similar to a cow´s mount. This Quanser platform allows us to quantify the error of the estimated angles of the IMU strapped down to the collar and to assess the energy used.

**Figure 1:**
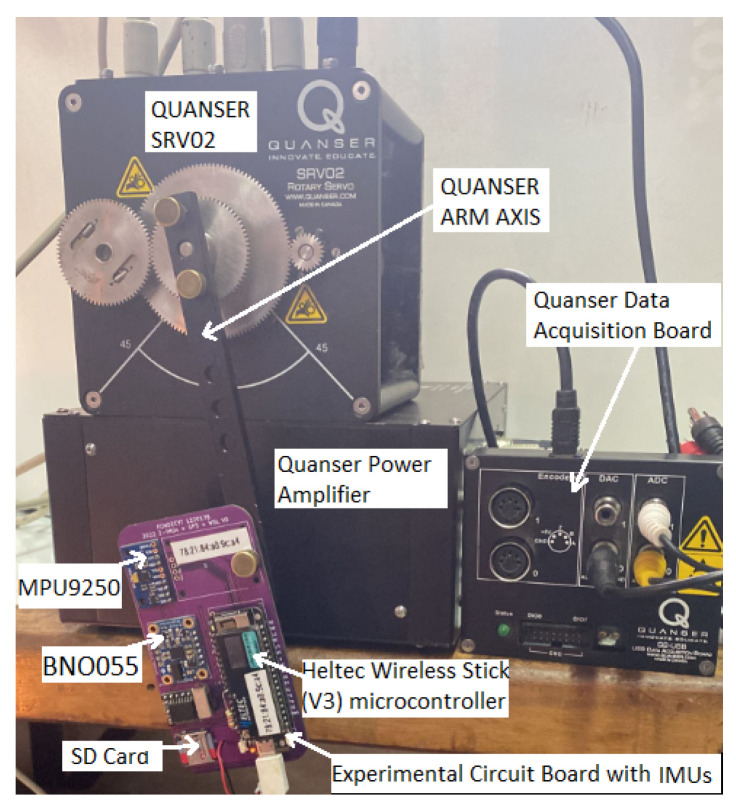
Experimental setup.

Figure 1 illustrates the experimental setup utilized in the design performed with the Quanser SRV02 [36] platform setup. The setup consists of a data acquisition module and a voltage-controlled linear power amplifier from the VoltPAQ to power the rotary servo base unit of the servo arm. This amplifier is explicitly designed to achieve a high performance in the hardware-in-the-loop (HIL) implementations. This platform reproduces movements with high repeatability, and uses accurate rotational angle sensors (0.2 degrees) as a reference signal. In the setup, the experimental board is attached to the arm’s end part of the platform SRV02 to retrieve the IMU signals while the platform arm is moving.

**Figure 2:**
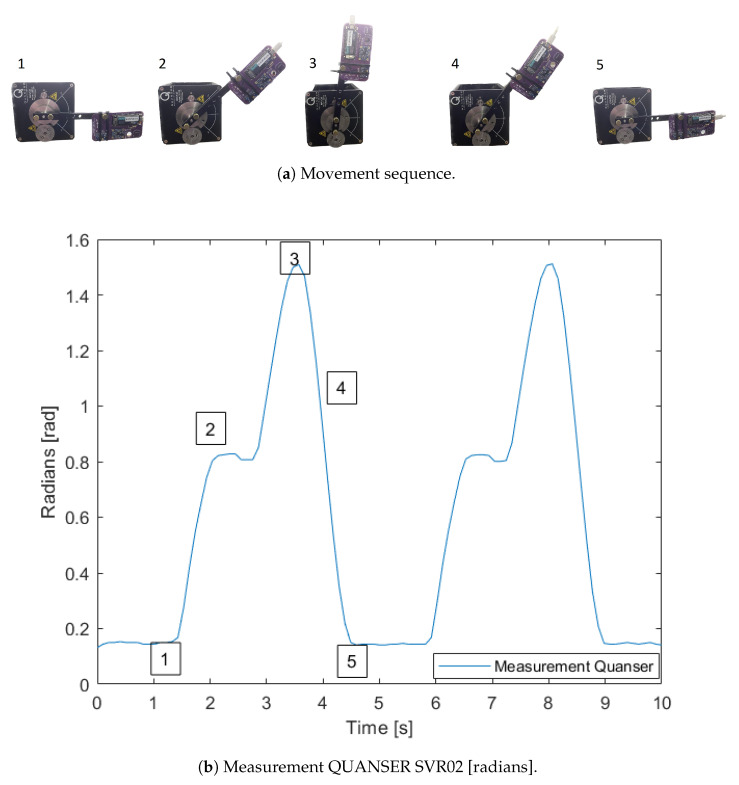
Test movements, (**a**) view of the movements at 5 waypoints, (**b**) measurement angles in Quanser SRV02.

For testing the algorithms, we will use the test curve shown in Figure 2b (radian units), which is expressed in Euler angles Θ=[ψ,θ,ϕ], and represents the cow mount movements. In the mount procedure, the cow’s initial position is over the rear end of another cow, and then it goes up, staying for a second, then it goes up again, and finally, it goes down. The test figure shows a 10-s-long test curve with the movement repeated twice. The figure shows only the pitch angle because this is the only angle of interest in the earth´s frame of reference, and the other two angles are kept almost constant.

In Equation (39), $J_{x}$ is an index of the error obtained in a reproduction of the test curve, *i* is the discrete-time at a rate of 10 samples per second, N=100 is the total samples in the curve, $\Theta_{m}$ is the reference attitude measured in the Quanser SRV02 with accurate shaft encoders. In (39), the attitude $\Theta_{m}$ is compared to five different test subjects $\Theta_{x}$, defined as follows:

(39) $$J_{x}=\sqrt{\frac{1}{N}\sum_{i=1}^{N}{\left(\Theta_{m}\left(i\right)-\Theta_{x}\left(i\right)\right)}^{2}}$$

- $\Theta_{x}=\Theta_{GI_{MPU}}$: the attitude in Euler angles computed only by integrating the gyroscope measurements provided by an InvenSense *MPU9250*. This quaternion is not self-calibrated;
- $\Theta_{x}=\Theta_{GI_{BNO}}$: the attitude in Euler angles computed only by integrating the gyroscope measurements provided by a BOSCH *BNO055*. This quaternion is not self-calibrated;
- $\Theta_{x}=\Theta_{SC_{MPU}}$: the attitude in Euler angles computed with the self-calibration method presented in Section 3, using the measurements (magnetometer, accelerometer, and gyroscope) provided by an InvenSense *MPU9250*;
- $\Theta_{x}=\Theta_{SC_{BNO}}$: the attitude in Euler angles computed with the self-calibration method presented in Section 3, using the measurements (magnetometer, accelerometer, and gyroscope) provided by a Bosch *BNO055*;
- $\Theta_{x}=\Theta_{Q_{BNO}}$: the attitude in Euler angles of the quaternions provided by the Bosch *BNO055*.

The test was conducted 200 times and the anomalous cases were excluded, resulting in a total of 180 valid cases. The choice of sample size was based on the results of Levene’s tests (p>0.544), which assessed the equality of variances. The procedure involved comparing variance across a range of experiments with different sample sizes, encompassing the values of N ranging from 30 to 200. The final conclusion was that the variance remains statistically constant from a sample size of 60 experiments onward as the sample size increases. However, due to the conditions of relative ease of experimental repeatability, a sample size of 200 experiments was chosen.

To create a more efficient and magnetometer-free routine, simplifying the automatic cow mount detection process. We employ a performance index similar to the one outlined in Equation (39) to assess our progress; however, we include a weighting matrix Λ. This matrix is essentially an identity matrix, but with the third component of the diagonal set to zero, allowing us to exclude errors associated with rotations around the *z* axis as follows:

(40) $$J_{Ix}=\frac{1}{N}\sum_{i=1}^{N}\left|\left|\Lambda \left[\Theta_{m}\left(i\right)-\Theta_{x}\left(i\right)\right]\right|\right|$$

- $\Theta_{x}=\Theta_{ISC_{MPU}}$: the attitude in Euler angles computed with the self-calibration method presented in Section 3.3, using only the accelerometer and gyroscope measurements provided by an InvenSense *MPU9250*;
- $\Theta_{x}=\Theta_{ISC_{BNO}}$: the attitude in Euler angles computed with the self-calibration method presented in Section 3.3, using only the accelerometer and gyroscope measurements provided by a BOSCH *BNO055*.

The parameters needed for the Kalman filter execution are the measurement variance, $R_{n}$, and the inner noise variance, $Q_{n}$. In this case, as the $R_{n}$ is a scalar value related to the measurement noise, the parameters were obtained with measurements taken from the IMU when it stood over a table for 15 min.

The parameters of the inner noise variance, $Q_{n}$, were estimated solving the problem as follows:

(41) $$Q_{n}=arg\min_{Q_{n}}\left\{\frac{1}{N}\sum_{i=1}^{N}\left|\left|\Theta_{m}\left(i\right)-\Theta_{x}\left(i\right)\right|\right|\right\}$$

We used the algorithm *fminsearch* of MATLAB with a data set resulting from a test using the Quanser SRV02 [36] involving 3200 s of data sampled at 10 Hz, and discarding the first 2 s to avoid the effect of the transient response of the Kalman filter (N=32,000).

### 4.3. Statistical Analysis Methods

We applied exhaustive statistical analyses to evaluate possible significant differences between the various orientation estimation methods. These tests begin with calculating descriptive statistics parameters and, subsequently, we performed normality tests using the indices obtained from Equation (39). These indices were computed based on the difference between three orientating estimation subject tests and the measured orientation performed in the Quanser SRV02 with the rotary encoders in the articulated arm. The three algorithms are the quaternions measurements provided by the *BNO055* and the self-calibrated algorithms based on the inertial sensors of the *MPU9250* and the *BNO055*. To validate the assumptions necessary for an ANOVA, we applied a normality test of the data obtained in Equation (39) with the Shapiro–Wilks. As the normality test failed, we used the Dwass–Steel–Critchlow–Fligner test as a non-parametric analysis to determine if there were significant differences between groups.

### 4.4. Energy Analysis

**Figure 3:**
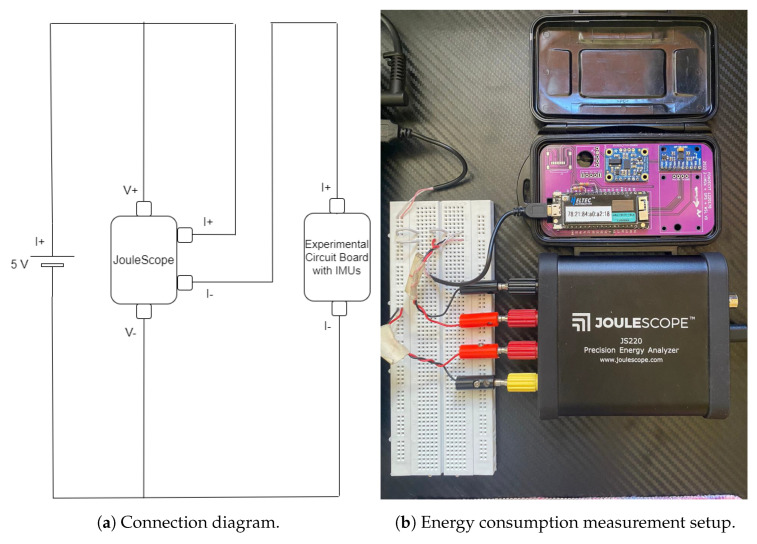
Experimental design for accurate measurement of electrical energy.

In this Section, we gauge the power increase incurred by the use of the attitude estimate routines presented in this article. To assess the power consumption, we used the Joulescope (see Figure 3b), which is a precision measuring instrument boasting a resolution of 0.5 nA and a bandwidth of 300 kHz, capable of handling 2 million samples per second [37]. The measurement circuit design is depicted in Figure 3a.

In this analysis, we identify the attitude estimate routines using lettered subscripts for differentiation, **A** refer to the measurement algorithms executed with data obtained from the *MPU9250*, while **B** is for those executed with data acquired from the *BNO055*. The subscripts indicate the following cases:

0. None of the algorithms were active, and this is for comparisons as a baseline. In this case, the IMU is powered, but in the configuration, the accelerometer, gyroscope, and magnetometer are disabled;
1. The simplified self-calibration algorithm (i.e., the inclinometer using only accelerometers and gyroscopes). In this case, only the magnetometer is disabled;
2. The comprehensive self-calibration algorithm considering all inertial measurements (accelerometer, gyroscopes, and magnetometers), and in the specific case of the *BNO055*;
3. The quaternion estimate provided by the Bosch algorithm in the *BNO055* IMU.

These details are summarized in Table 1. The Joulescope measurements focus on energy consumption, expressed in milliwatts, [mW]. The experiments involve the individual execution of each self-calibration algorithm over a 10-min interval with a sample time of 0.1 s. Subsequently, statistical metrics, such as mean and standard deviation, are calculated. Additionally, normality tests will be conducted, followed by parametric and non-parametric statistical tests, depending on the data’s nature. *JAMOVI* statistical software [38] are for determining the presence of significant differences.

The choice of a 10 min interval for each execution is justified regarding the stability and consistency in measurements over time. Additionally, it is taken into account that the Levene test (p>0.279) for the equality of variances is assumed after the first 5 min, ensuring that the required conditions for this analysis are properly met.

## 5. Results

### 5.1. Initial Calibration of the Accelerometer for MPU9250 and BNO055

Table 2 describe the calibration results of the accelerometer model parameters using Equations (4) and (5) for a set of 30 accelerations measured at orientations obtained when the three rotation axis were changed at regular intervals.

### 5.2. Initial Calibration of the Magnetometer for *MPU9250* and *BNO055*

Table 3 describe the calibration results of the magnetometer model parameters using Equations (9) and (10) for a set of 30 magnetometer measurements acquired when the 3 rotation axis were modified at regular intervals.

### 5.3. Results of Self-Calibration Algorithms

The results of the optimization problem (41) applied to the extended Kalman filter for *MPU9250* and *BNO055* are provided below for the estimation of $Q_{n}$. It is worth noting that this routine was specifically employed to compute error parameters associated with $Q_{n}$, while the matrix $R_{n}$ was directly measured and given by the following:

$$\begin{array}{cc} Q_{MPU} & =\mathrm{diag}\left([1.2681,\;3.8625,\;4.5505,\;9.5457,\;0.0670,\;0.0893,\;0.0292]\right)10^{-5}\\ R_{MPU} & =\mathrm{diag}\left([0.0011,\;0.0026,\;0.0031,\;0.0012,\;0.0026,\;0.0010]\right)\\ Q_{BNO} & =\mathrm{diag}\left([8.9884,\;2.4526,\;4.4477,\;3.4589,\;0.0227,\;0.0105,\;0.0186]\right)10^{-6}\\ R_{BNO} & =\mathrm{diag}\left([0.0011,\;0.0016,\;0.0024,\;0.0227,\;0.0262,\;0.0276]\right) \end{array}$$

**Figure 4:**
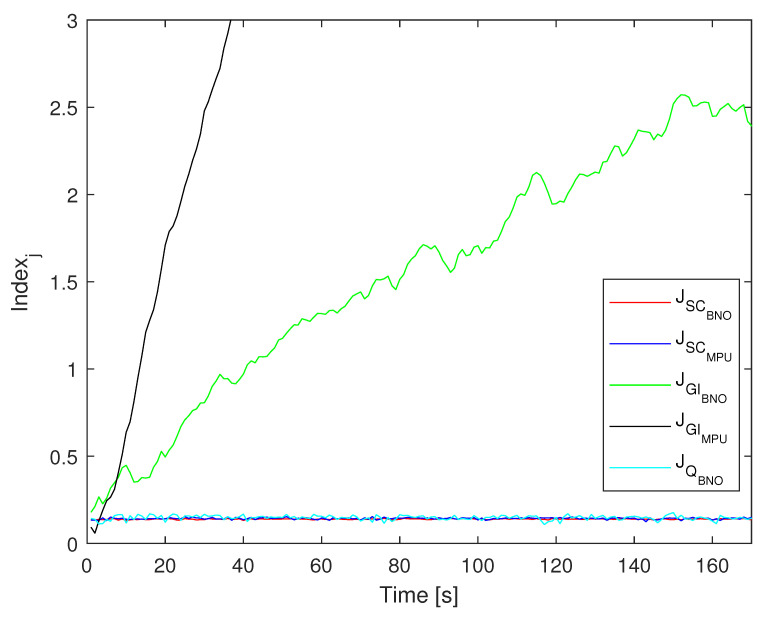
Evolution of the error index $J_{x}$ for algorithms $J_{SC_{MPU}}$, $J_{GI_{MPU}}$, $J_{SC_{BNO}}$, $J_{GI_{BNO}}$ and $J_{Q_{BNO}}$.

Figure 4 illustrates the evolution of measurement indices assessed through the five proposed algorithms. As anticipated, the methods that integrate angular velocity from the *MPU9250* and *BNO055* IMUs demonstrate poor performance over the extended integration duration. This observed behavior renders the integration of angular velocity impractical for collar-worn devices intended to analyze the acceleration in terms of the resulting angles used as matrix rotations between the body and world frames. The other algorithms’ key disparity between these two algorithms lies in the substantially higher error due to the bias $\bar{b}$ of the gyroscopes.

Compared to the other proposed algorithms, which share similar characteristics by maintaining a relatively constant mean error with variations attributed to random variables, the Kalman filter method that utilizes the *MPU9250* stands out for its ability to provide a smaller margin of error.

### 5.4. Statistical Results

Table 4 shows the results of comparing the means, $\mu (J_{x})$, and standard deviation, $\sigma (J_{x})$, for each test subject (39) when the test corresponds to similar mount movements (see Figure 2b) was repeated 185 times in the *SRV02* platform. The lower result, as expected, is the obtained one with the attitude quaternion computed only by integrating the gyroscope measurements provided by an InvenSense *MPU9250* and a Bosch *BNO055*, represented by $J_{GI_{MPU}}$ and $J_{GI_{BNO}}$. These quaternions are not self-calibrated, and the high mean value of $J_{MPU}$ is due to the variability of the bias. The other three indices are very close, meaning the error is less significant when using either technology. In Table 3, the best results were obtained with the self-calibrating algorithm and the InvenSense *MPU9250*, a low-cost IMU. The self-calibrating algorithm applied to the Bosch *BNO055* shows better performance with this index than the quaternion provided in this IMU. The results of the normality tests using the Shapiro–Wilks criteria applied to the indices indicate that with 95% confidence, the proposed orientation estimation algorithms do not follow a normal distribution (p<0.001).

**Figure 5:**
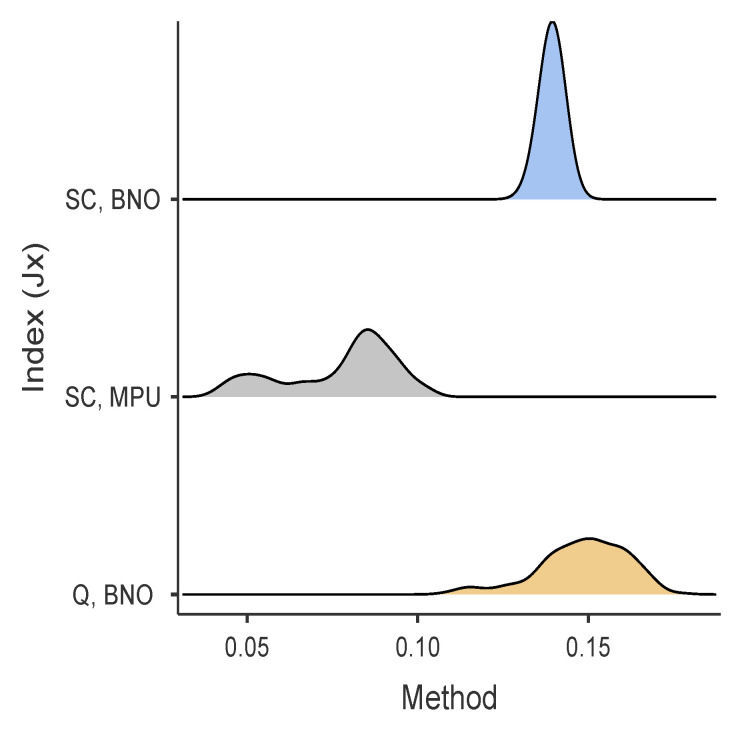
Error distribution density of Kalman for self-calibration using *BNO055* ($J_{SC_{BNO}}$) and *MPU9250* ($J_{SC_{MPU}}$), and the quaternion estimates provided by Bosch in the *BNO055* ($J_{Q_{BNO}}$).

Figure 5 illustrates the distribution density of the error index using Kalman filtering and quaternion measurements from the *BNO055*. It is evident that the curves for *MPU9250* and *BNO055* are similar, with MPU having a slight edge, but both are significantly different on average from the quaternions obtained from the *BNO055* (Kruskal–Wallis p<0.001). Furthermore, using the statistical software *JAMOVI* [38], we estimate the effect size, Cohen’s d, ($\epsilon^{2}$ = 0.89), which is considered a large effect according to Cohen’s standards.

### 5.5. Results of Inclination Auto-Calibration

**Figure 6:**
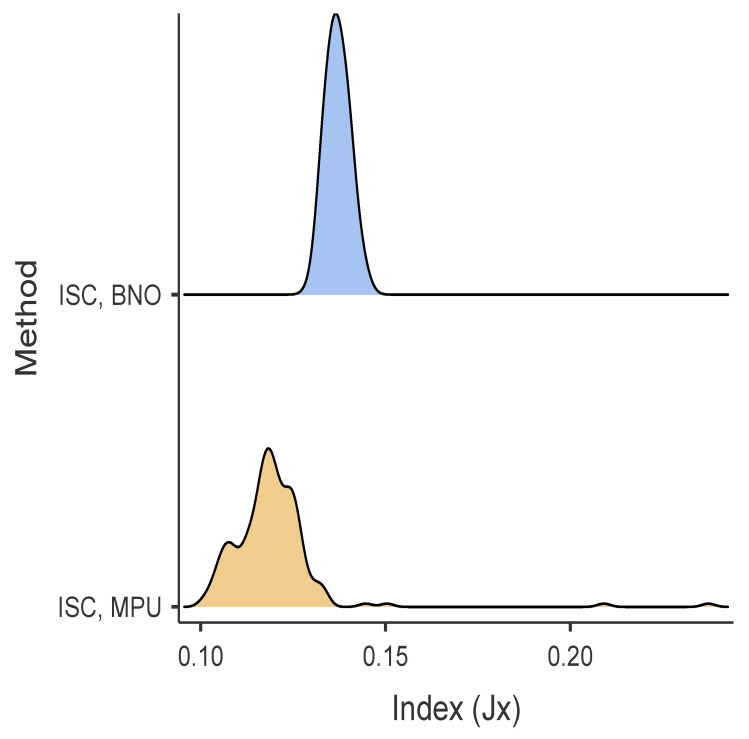
Error distribution density of Kalman filter for inclination self calibration using *BNO055* ($J_{x}=J_{ISC_{BNO}}$) and *MPU9250* ($J_{x}=J_{ISC_{MPU}}$).

The descriptive statistical analysis results suggest that the mean of the MPU Kalman filter is slightly superior to that of the *BNO055* Kalman filter, while the *BNO055* exhibits a lower estimation variability. Normality tests at a 95% confidence level fail to provide sufficient evidence for the normality of the results (see Table 5). Consequently, a non-parametric Kruskal–Wallis test was conducted to assess the differences in outcomes between both algorithms. The results indicate the presence of statistically significant mean differences (p<0.001). Figure 6 depicts that the error distribution of the algorithm with the Kalman filter based on the measurements of the *MPU9250* is lower than that obtained with the Kalman filter based on the measurements of the *BNO055*. Furthermore, the results using the statistical software *JAMOVI* [38] estimate the effect size, Cohen’s d, ($\epsilon^{2}$ = 0.696), which is considered a large effect according to Cohen’s standards [39].

### 5.6. Result Energy Analysis

**Figure 7:**
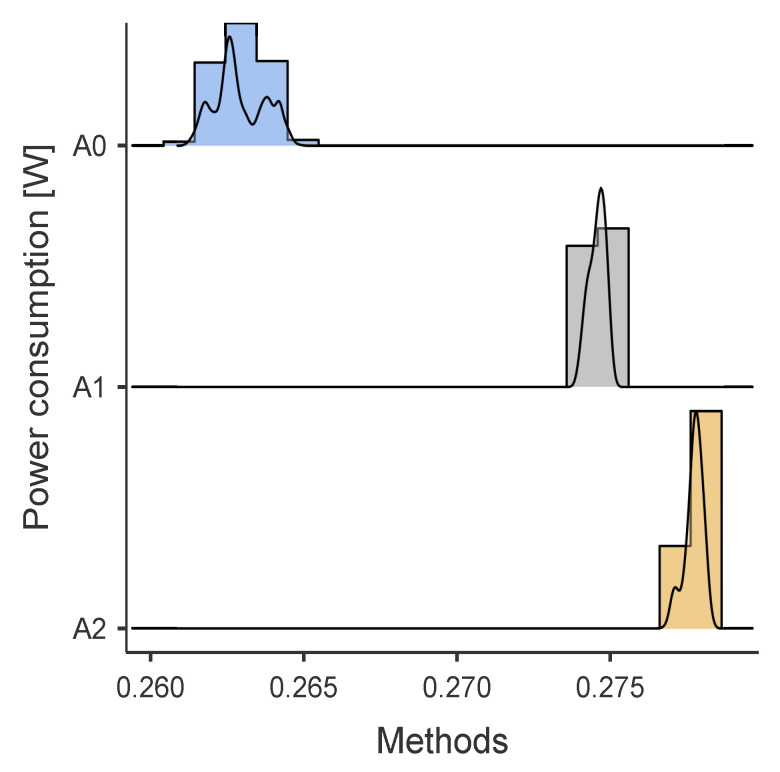
Energy consumption results $A_{0}$, $A_{1}$, and $A_{2}$ with Invensense *MPU9250*. Methods $A_{0}$: *MPU9250* energized but inactive, *BNO055* not connected. Methods $A_{1}$: inclination-self-calibration algorithms with data acquired from *MPU9250*. Methods $A_{2}$: Self-calibration algorithm with data acquired from *MPU9250*.

**Figure 8:**
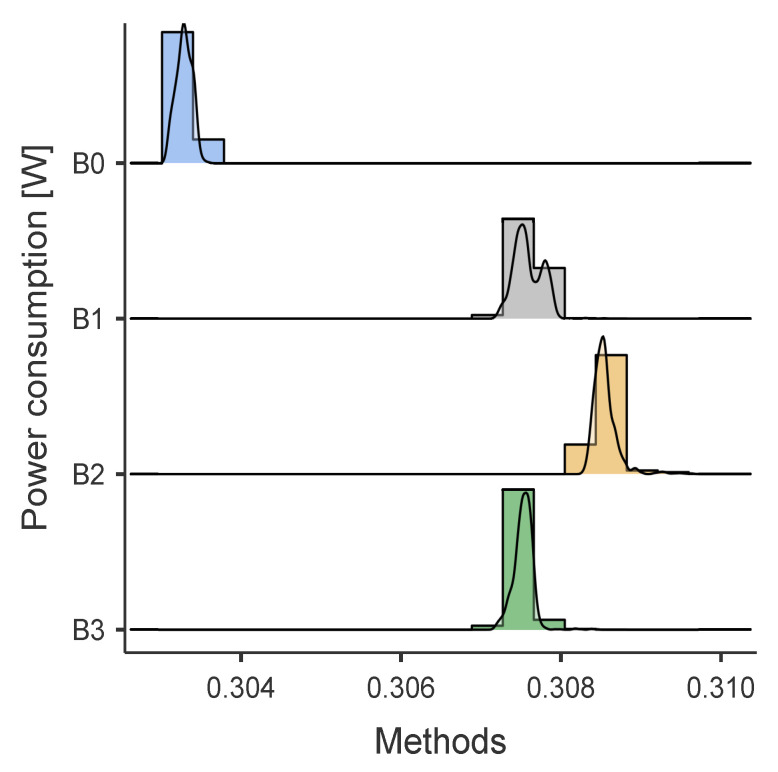
Energy consumption results $B_{0}$, $B_{1}$, $B_{2}$, and $B_{3}$ with Bosh *BNO055*. Methods $B_{0}$: *BNO055* energized but inactive, *MPU9250* not connected. Methods $B_{1}$: inclination-self-calibration algorithm with data acquired from *BNO055*. Methods $B_{2}$: self-calibration algorithm with data acquired from *BNO055*. Methods $B_{3}$: quaternion attitude estimates from *BNO055*.

Table 6 resumes the obtained power consumption results [mW] obtained in the tests described in Section 4.4. Figure 7 and Figure 8 show the power consumption distributions for *BNO055* and *MPU9250*, respectively. Furthermore, the results using the statistical software *JAMOVI* [38] of the non-parametric tests indicate that there are statistically significant differences between the algorithms for the *BNO055* and *MPU9250* (p<0.00001) and effect size estimates, Cohen’s d, ($\epsilon^{2}$ = 0.8538) for *BNO055* and ($\epsilon^{2}$ = 0.8881) for *MPU9250*, which is considered a large effect according to Cohen’s standards.

## 6. Discussion

This study showed that a simple but effective method implemented in a low-cost microcontroller could accurately estimate the device’s attitude with an IMU strapdown when stressed in similar conditions to a cow collar. For this work, we assumed that the acceleration resulting from the cow’s movement is negligible. Yet, it remains present, influencing the inclination due to acceleration when the cow is in motion, while the errors highlighted in this study are not deemed significant, assessing the potential impact of these inclination errors on classification algorithms is relevant. Moreover, a thorough examination of the impact of inclination errors on classification algorithms could enlighten future research on enhancements for the auto-calibration algorithm.

Incorporating self-calibration algorithms, such as those based on Kalman filters, into previous works on motion classification using IMUs can have considerable effects on the precision and efficiency of said classifications. By addressing the challenges associated with orientation variability and the need for precise calibration, these algorithms could significantly improve the robustness of classification models, allowing for a more accurate interpretation of motion patterns.

Variability in the orientation of the IMUs during data capture can influence the results of motion classification. The addition of self-calibration algorithms for slow-moving bodies, such as a cow, helps mitigate this issue, providing more consistent results regardless of device’s position. The use of self-calibration algorithms would reduce this dependency, facilitating the transition between different IMU providers without the need for significant adjustments.

The successful incorporation of self-calibration algorithms in previous works highlights the need for future research that explores the adaptability and performance of these algorithms in various contexts and specific applications, thus allowing continuous evolution in the improvement of IMU-based technologies.

Sensor choice and energy efficiency are critical factors in device life and performance. Implementing self-calibration algorithms can enable a more flexible selection of sensors, thus optimizing power consumption and extending battery life. The energy analysis for the *MPU9250* and the *BNO055* (see Table 6) highlights differences between the power consumption requirements of IMUs with a potential impact on battery life. Thus, this analysis provides a practical perspective focused on determining the relationship between the accuracy of orientation estimation measurements and energy efficiency. Although the inclination self-calibrating algorithm offers only accurate orientation information with respect to the *z-axis*, they use 3.1 mW less energy for *MPU9250* and 0.9 mW less for *BNO055* with respect to the full calibration. These differences are significant when we project the energy consumption along the life cycle of the IoT Collar.

## 7. Conclusions

The innovation of this work is the use of strapdown inertial navigation systems as an autonomous, accurate method for estimating cattle orientation, emphasizing its impact on long-term power efficiency, the benefit for animal behavior monitoring and energy-efficient field applications, and advancing the understanding of cattle behavior. The proposed method was experimentally verified using an arm axis that generates rotational movements similar to a cow and executing a statistical analysis of performance and a power analysis of the developed system.

In this paper, we developed two versions of the Kalman filter to estimate the attitude of an IMU strapped to a cow’s collar. The first version used a correction based on acceleration and magnetometer measurements. The resulting algorithm corrects the bias entirely and is very useful for supporting the identification of cow movements. The other one uses only the accelerometers, which partially corrects bias. In this case, the bias associated with the tilt and roll angles is accurate, but the bias associated with the rotation in the z-axis has a drift. This algorithm is still helpful to support identifying some cow movements, particularly those associated with mounts and walk.

The quaternion Kalman filter for the auto-calibration of the attitude is simple enough to allow coding into the microcontroller installed on the cow collar. Although the attitude quaternion corresponds to a rotation, it is advisable to include a correction between iterations to maintain their module equal to one. The algorithm assumes that most of the time, the acceleration produced by the cow is negligible compared with the acceleration of gravity, the attitude estimation shows good results, and due to its simplicity, the algorithm has a low demand for computing, maintaining a low energy load to diminish the collar’s battery drain. This is a promising result to continue developing classification algorithms based on attitude estimates to execute them at the edge in the cow’s IoT collar using low-cost IMUs. Further work must evaluate the impact of the attitude estimate error on the classification when identifying different cow movements, such as mounts, regular walking, and gait.

The proposal to use self-calibration algorithms using Kalman filters for IoT cow collars seeks to look for guidance to acquire correct acceleration measurements in a common reference frame. These algorithms have a slight impact on processing time and energy consumption. However, the choice of sensor is crucial in terms of energy efficiency and presents significant differences. Notable discrepancies were found in average power consumption, with a difference of around 30 mW between the *MPU9250* and the *BNO055*. This disparity is considerable in terms of duration, highlighting the importance of selecting the appropriate sensor.

To advance in searching for SINS to support the rotation of coordinates in a cow collar with low power requirements, accuracy, and computing load, we must keep exploring the algorithms’ efficiency in various IMUs and trying algorithm alternatives to the Kalman filter, such as the Complimentary filter [40] or the Madgwick filter [41]. Future research should investigate the synergies and potential improvements that arise from integrating these filters, providing a comprehensive assessment of its impact on classification accuracy for various cow movements, including riding, regular walks, and walking.

We searched for simple algorithms to be included in cow collars to empower the cow collar with SINS. The proposed algorithms perform a real-time gyro bias calibration and attitude estimation. Initially, we manually calibrated the accelerometer and magnetometer to have a fair comparison. This initial calibration is complex because we must orient the device in several positions. Once the collars will be installed on the cows, we cannot recalibrate the accelerometer and the magnetometer, while the accelerometers’ scaling, alignment, and bias parameters do not change significantly with time, the anomalies affecting the parameters of the magnetometers are more significant, deteriorating not only the yaw update but also the roll and pitch estimate. The Kalman filter with measurement updates based only on accelerations presented in Section 2.3 shows a reasonable tilt estimate. Applying this rotation on the body axes produces a representation that favors the discrimination between horizontal and vertical movements. This algorithm is more straightforward, as it does not use the magnetometer and consumes less power; thus, we recommend using this algorithm for running at the edge in the microcomputers of the cow collar.

## Figures and Tables

**Table 1:** Experimental description.

Algorithm Types	Description
$A_{0}$	Base line for *MPU9250*
$A_{1}$	Algorithms “ISC” for *MPU9250*
$A_{2}$	Algorithms “SC” for *MPU9250*
$B_{0}$	Base line for *BNO055*
$B_{1}$	Algorithms “ISC”s for *BNO055*
$B_{2}$	Algorithms “SC” for *BNO055*
$B_{3}$	Algorithms “Q” for *BNO055*

SC: self-calibration; ISC: inclination self-calibration; *Q*: quaternion native estimation.

**Table 2:** Calibrated parameters of the accelerometer, $\theta_{a}$, for *MPU9250* and *BNO055* IMU’s. Source: self-made.

Parameter [g=9.8 m/s^2^]	Invensense *MPU9250*	Bosch *BNO055*
$\gamma_{a,xz}$	0.0016	2.9621 $\times \;10^{-5}$
$\gamma_{a,xy}$	−0.0017	−8.5622 $\times \;10^{-5}$
$\gamma_{a,yx}$	0.009	−4.15645 $\times \;10^{-5}$
$\gamma_{a,yz}$	0.0091	−2.13033 $\times \;10^{-5}$
$\gamma_{a,zy}$	0.0060	−2.6978 $\times \;10^{-5}$
$\gamma_{a,zx}$	−0.0009	−7.82731 $\times \;10^{-5}$
$K_{a,x}$	0.0615	0.0010360
$K_{a,y}$	0.0610	0.0010356
$K_{a,z}$	0.0601	0.0010321
$b_{a,x}$	219.8976	8.3184
$b_{a,y}$	287.6430	−21.9462
$b_{a,z}$	1346.7	−8.5860

**Table 3:** Table of calibrated magnetometer parameters, $\theta_{b}$, for *MPU9250* and *BNO055* IMU’s. Source: self-made.

Parameter [μT]	Invensense *MPU9250*	Bosch *BNO055*
$\gamma_{m,xz}$	0.000414	1.2614 $\times \;10^{-5}$
$\gamma_{m,xy}$	−0.0003	−2.9753 $\times \;10^{-5}$
$\gamma_{m,yx}$	−0.0001	−2.1678 $\times \;10^{-5}$
$\gamma_{m,yz}$	0.0032	4.3141 $\times \;10^{-5}$
$\gamma_{m,zy}$	0.00015	−1.4241 $\times \;10^{-5}$
$\gamma_{m,zx}$	0.00041	−2.6346 $\times \;10^{-5}$
$K_{m,x}$	0.02844	0.0011077
$K_{m,y}$	−0.0284	0.0011077
$K_{m,z}$	0.02752	0.0011166
$b_{m,x}$	−22.18170	−3.832519
$b_{m,y}$	5.3160	−0.120414
$b_{m,z}$	−44.9796	9.245934

**Table 4:** Mean and standard deviation of the error indices when the test curve was executed 185 times.

Metrics	$J_{{\mathrm{SC}}_{\mathrm{MPU}}}$	$J_{{\mathrm{SC}}_{\mathrm{BNO}}}$	$J_{Q_{\mathrm{BNO}}}$	$J_{{\mathrm{GI}}_{\mathrm{BNO}}}$	$J_{{\mathrm{GI}}_{\mathrm{MPU}}}$
μ	0.0774	0.1393	0.1493	1.6717	8.5258
σ	0.0164	0.0024	0.0130	0.7142	5.0098
Shapiro–Wilks	<0.001	<0.001	<0.001	<0.001	<0.001

$J_{SC_{BNO}}$: self-calibration with data from *BNO055*; $J_{SC_{MPU}}$: self-calibration with data from *MPU9250*; $J_{GI_{BNO}}$: gyroscope integration with data from *BNO055*; $J_{GI_{MPU}}$: gyroscope integration with data from *MPU9250*; $J_{Q_{BNO}}$: native quaternion from *BNO055*.

**Table 5:** Mean and standard deviation of the error indices when the test curve was executed 185 times.

Metrics	$J_{{\mathrm{ISC}}_{\mathrm{MPU}}}$	$J_{{\mathrm{ISC}}_{\mathrm{BNO}}}$
μ	0.1195	0.1370
σ	0.0135	0.0032
Shapiro–Wilks	<0.001	<0.001

$J_{ISC_{MPU}}$: inclination self-calibration with data from *MPU9250*; $J_{ISC_{BNO}}$: inclination self-calibration with data from *BNO055*.

**Table 6:** Mean and standard deviation of energy consumption.

		*MPU9250*				*BNO055*	
Method	$\bar{x}\left[mW\right]$	σ[mW]	Shapiro–Wilk	Method	$\bar{x}\left[mW\right]$	σ[mW]	Shapiro–Wilk
$A_{0}$	262.9	0.8	<0.00001	$B_{0}$	303.3	0.1	<0.00001
$A_{1}$	274.6	0.3	<0.00001	$B_{1}$	307.6	0.2	<0.00001
$A_{2}$	277.7	0.3	<0.00001	$B_{2}$	308.5	0.2	<0.00001
-	-	-	-	$B_{3}$	307.5	0.1	<0.00001

## Data Availability

Data are contained within the article.

## Glossary and Abbreviations

The following abbreviations are used in this manuscript:

SINS	Strapdown inertial navigation system
SC	Self-calibration
ISC	Inclination self-calibration
$a_{c}$	Calibrated accelerometer
$a_{m}$	Accelerometer measurement
$K_{a}$	Scaling matrix for accelerometer
$s_{a}$	Non-orthogonal matrix for accelerometer
$b_{a}$	Bias for accelerometer
$n_{a}$	Noise for accelerometer
$B_{c}$	Calibrated magnetometer
$B_{m}$	Magnetometer measurement
$K_{b}$	Scaling matrix for magnetometer
$s_{b}$	Non-orthogonal matrix for magnetometer
$b_{b}$	Bias for magnetometer
$n_{b}$	Noise for magnetometer
$J_{GI_{BNO}}$	Performance error of gyroscope integration with data from *BNO055*
$J_{GI_{MPU}}$	Performance error of gyroscope integration with data from *MPU9250*
$J_{SC_{MPU}}$	Performance error of self-calibration with data from *MPU9250*
$J_{SC_{BNO}}$	Performance error of self-calibration with data from *BNO055*
$J_{Q_{BNO}}$	Performance error of native quaternion from *BNO055*
$J_{ISC_{MPU}}$	Performance error of inclination self-calibration with data from *MPU9250*
$J_{ISC_{BNO}}$	Performance error of inclination self-calibration with data from *BNO055*
